# Rapid change of limiting nutrient in the Gulf of Bothnia, northern Baltic Sea

**DOI:** 10.1038/s41598-026-69219-6

**Published:** 2026-09-04

**Authors:** Siv Huseby, Agneta Andersson, Karolina Ida Anna Eriksson, Sonia Brugel, Joakim Ahlgren

**Affiliations:** 1 Umeå Marine Sciences Center, Umeå University, Umeå, Sweden; 2 Department of Ecology, Environment and Geosciences, Umeå University, Umeå, Sweden

**Keywords:** Gulf of Bothnia, Northern Baltic Sea, Dissolved inorganic nitrogen, Dissolved inorganic phosphorus, Limiting nutrient, Nutrient stoichiometry, Biogeochemistry, Ecology, Ecology, Environmental sciences, Ocean sciences

## Abstract

Historically, phosphorus has been considered the limiting nutrient for primary production in the northern Baltic Sea, whereas further south in the Baltic Proper nitrogen is the limiting nutrient. In this study, we investigated the nutrient distribution during the past 30 years using Swedish and Finnish monitoring data. The results show comprehensively for the first time that the entire Bothnian Sea has switched to nitrogen limitation and that the Bothnian Bay is rapidly heading towards similar conditions. This is due to increasing inorganic phosphorus concentrations, possibly resulting from phosphorus rich water inflowing from the Baltic Proper. Inorganic nitrogen concentrations decrease or are unchanged. The increase of phosphorus led to increased primary production and, in the Bothnian Sea, to increased occurrence of filamentous cyanobacteria. The resulting shift emphasizes the need to revise management strategies of these basins, where treating both basins for phosphorus and nitrogen is advisable.

**Supplementary Information:**

The online version contains supplementary material available at https://doi.org/10.1038/s41598-026-69219-6.

## Introduction

Eutrophication, as an excessive nutrient richness causing increased algal growth, is one of the major environmental problems in the Baltic Sea. The most recent assessment, HELCOM HOLAS 3, covering the period 2016–2021, concluded that at least 93% of the region has moderate or poor environmental status concerning eutrophication^1^. This is a slight improvement compared to the previous 5-year assessment from 2011 to 2016 which stated that 97% of the Baltic Sea was affected by eutrophication^2^. The environmental measures enforced, including those of the Baltic Sea Action Plan that was adopted in 2007, thus seem to have some positive ecosystem effect on the Baltic Sea as a whole.

In the HELCOM assessment, the concentrations of nitrogen and phosphorus are evaluated and used to assess the eutrophication status in different areas of the Baltic Sea. It has been debated heavily which of the two nutrients, nitrogen or phosphorus, limits primary production and acts as the driver of eutrophication^3^. Which nutrient limits production have implications for ecosystem productivity and food web quality^4,5^ and is therefore of great importance also for water management agencies. In a broader context within ecological stoichiometry, availability of other essential elements than nitrogen and phosphorus has been shown to have implications for productivity in marine ecosystems^6^. Understanding effects of other elements, including the association between them, and other conditions like temperature on aquatic ecosystems is steadily growing^7^. An example of this is the increased understanding of how the ecosystem of the Gulf of Bothnia is shaped by riverine inputs^8^ through the association between nitrogen and carbon and its implications for production and efficiency of the ecosystem^9^. Within governance of the Baltic Sea, however, most focus is still on the main nutrients, nitrogen and phosphorus, when adopting measures combating eutrophication.

The Gulf of Bothnia is the collective name of the northernmost part of the Baltic Sea and consists of two basins: the Bothnian Bay in the north and the Bothnian Sea in the south, separated by a sill. Historically, this sea area has not been considered affected by eutrophication^10,11^, but in the most recent assessment, the nutrient levels and the indicators for direct effects of eutrophication, such as chlorophyll *a* concentrations and cyanobacteria bloom index, show poor or moderate status for both the Bothnian Bay and the Bothnian Sea^1^. The primary production in the Baltic Proper is in general regarded as nitrogen limited^6,8,10,12^, while the Bothnian Bay has been described as mainly phosphorus limited^10^. A few observations of nitrogen limitation have been made on the eastern coast of the Bothnian Bay^11^, indicating spatial variation in the basin, but studies on both spatial and temporal variations are largely lacking in the Bothnian Bay.

The Bothnian Sea has been considered as a transition zone between the nitrogen limited Baltic Proper and the phosphorus limited Bothnian Bay^13,14^. There is, however, growing evidence that nitrogen is the limiting nutrient for primary production in this basin^10,11,15–17^, although only a few studies state this firmly. There is also emerging evidence of eutrophication effects in the Bothnian Sea with increasing duration and spatial coverage of cyanobacteria blooms^18^ and increasing biomass of diazotrophic filamentous cyanobacteria^4,19^. Decreasing oxygen concentrations in the bottom waters are also observed, mainly likely due to inflow of waters from the Baltic Proper with low oxygen concentrations in combination with higher temperatures^20^.

The increasing evidence of eutrophication in the northern Baltic Sea^1^, combined with the conflicting historical views regarding limiting nutrient in the different parts of the Gulf of Bothnia, points to the necessity of a more extensive look at the nutrient distribution in the entire northern Baltic Sea. This has also been an apparent need of environmental governance agencies in Sweden both on a regional and a national level, especially related to the revision of the European Wastewater Directive 2024/3019^21^. In southern and middle Sweden, sewage treatment plants reduce both phosphorus and nitrogen, as the Baltic Proper is nitrogen limited, while in northern Sweden, as well as in Finland, only phosphorus is reduced up until the revised directive 2024, since the Gulf of Bothnia has been considered not to be nitrogen sensitive. This alleviation in the wastewater treatment procedure may need to be changed if all parts of the Gulf of Bothnia are clearly shown to be nitrogen sensitive.

The aims for this study were therefore to elucidate (1) if and how the inorganic phosphorus and nitrogen concentrations have changed in coastal and offshore areas in the Bothnian Sea and the Bothnian Bay during the past 30 years, and (2) if the nutrient stoichiometry and most limiting nutrient have changed. The study was carried out by analyzing Swedish and Finnish time series monitoring data, and by performing nutrient addition experiments to assess the boundaries between nitrogen and phosphorus limitation for primary production. This study thus concludes the first comprehensive review of all available nutrient data in the Gulf of Bothnia, and the first assessment of changes in the limiting nutrient and its consequences.

### Study area

**Fig. 1:**
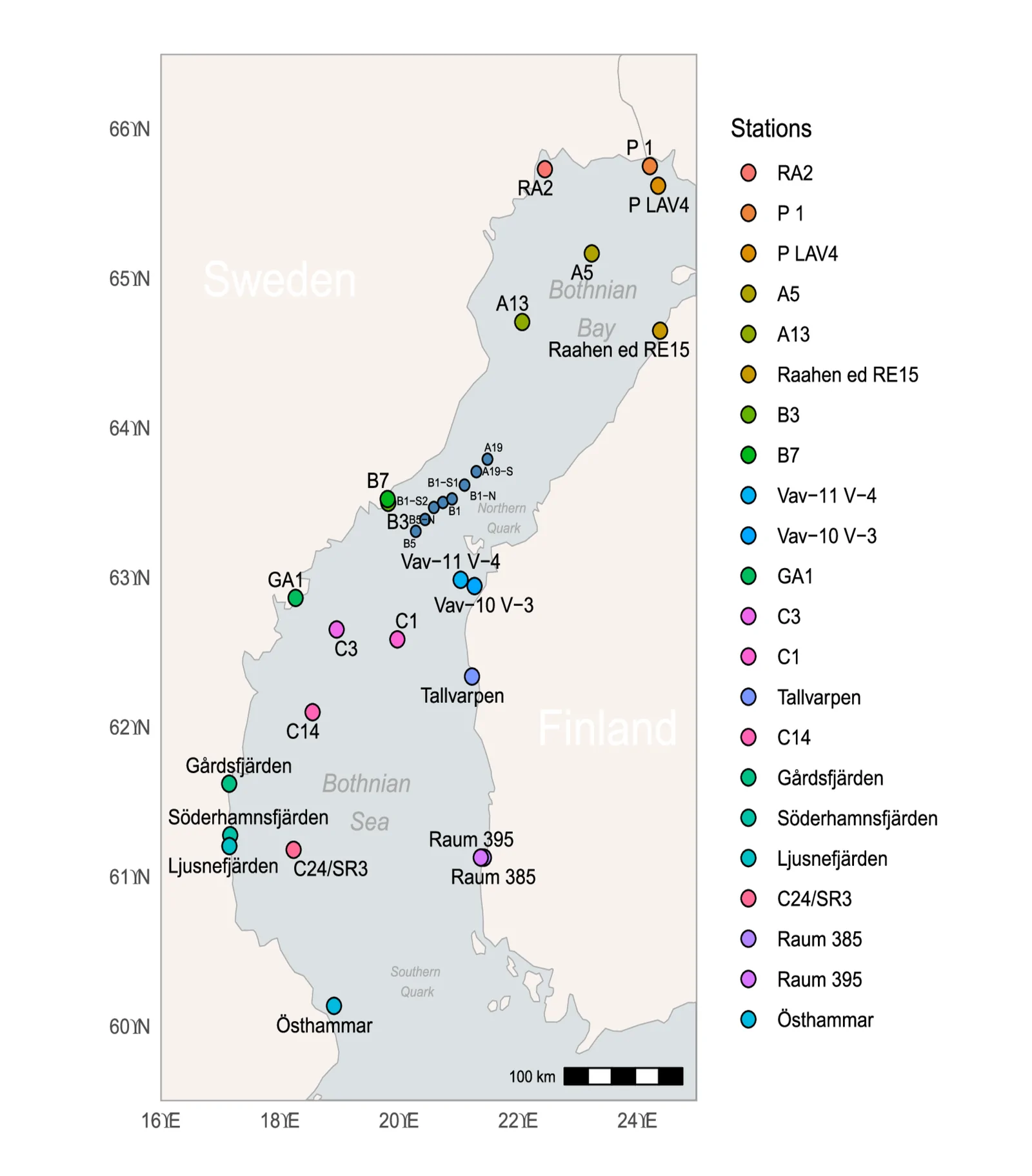
Map of the study area showing monitoring stations from where data were used for analysis and stations sampled for the nutrient limitation experiment. Map made using R, version 4.6.1, (https://cran.r-project.org/bin/windows/base/old/4.6.1/) and the following R packages: tidyverse version 2.0.0^27^, dplyr version 1.2.1^28^, ggplot2 version 4.0.3^29^, rnaturalearth version 1.2.0^30^, rnaturalearthdata version 1.0.0^31^, ggspatial version 1.1.10.^32^.

The study area shows a strong salinity gradient with lower salinities in the north. The two basins are separated by the shallow northern Quark area (Fig. 1). In the south, there is a similar shallow area, the southern Quark, which separates the Bothnian Sea from the Baltic Proper.

The Bothnian Bay has a low salinity, ranging from 1 to 4 PSU. Both coastal and offshore areas in the Bothnian Bay are ice covered typically during the period December-April, although the extension of the ice cover is decreasing as a result of warmer climate, where the largest ice-cover reduction is found in the southern areas^22,23^. The basin is strongly influenced by freshwater runoff from several large rivers, which apart from giving low salinities also discharge organic matter from the surrounding drainage area.

The Bothnian Sea has a higher salinity than in the Bothnian Bay, ranging from 4 to 7 PSU. Although along the coast many areas are highly influenced by riverine inflow, the basin is not as influenced by freshwater run-off as the Bothnian Bay.

## Materials and methods

### Analysis of monitoring data

Nutrient data were mainly gathered through the Swedish national database SHARKweb (https://sharkweb.smhi.se/) provided by the Swedish Meteorological and Hydrological Institute (SMHI) and the Finnish database Merihavainnot.fi provided by the Finnish Environment Institute (SYKE). In addition to this, data were also gathered through the database dBotnia at Umeå Marine Sciences Center (UMSC), Umeå University, as well as from various local and regional recipient control programs. Information on the analytical and sampling methods used for data collection can be found in the database documentation. For the HELCOM area, most national monitoring programs follow the methods described in the HELCOM Combine guidelines (https://helcom.fi/helcom-at-work/publications/manuals-and-guidelines/). For nutrient analysis, the reference method used is mainly Grasshoff^24^.

Data for the years 1994–2023 were aggregated as yearly winter means for the months December to March, for 0–10 m of depth. Data for time series analysis were mainly aggregated station wise, using stations from the national monitoring programs of Sweden and Finland as shown in Fig. 1, with at least 10 years of consecutive data. Stations shown in the map are grouped in 6 regional groups in the results section; Swedish coast of Bothnian Bay (station RA2), Finnish coast of Bothnian Bay (stations P1, P LAV 4 and Raahen ed RE15), Offshore Bothnian Bay (stations A5, A13), Swedish coast Bothnian Sea (B3, B7, GA1, Gårdsfjärden, Söderhamnsfjärden, Ljusnefjärden and Östhammar), Finnish coast Bothnian Sea (stations Vav-11 V-4, Vav 10 V-3, Tallvarpen, Raum 385 and Raum 395) and Offshore Bothnian Sea (stations C1, C3, C14 and C24/SR3). Data for spatial analysis was aggregated to regional water bodies using data from both national and regional monitoring and plotted in maps using QGIS, version 3.30.2 (https://qgis.org).

To investigate the limiting nutrient the Redfield ratio was used. This is the most common method to determine the limiting nutrient due to the constant molar ratio composition at 16:1 for nitrogen relative phosphorus in a wide range of phytoplankton^25^. Thus, the ratio between dissolved inorganic nitrogen (DIN, sum of ammonia, nitrite, and nitrate) and dissolved inorganic phosphorus (DIP, phosphate) was calculated for all investigated sampling stations, water bodies and time periods. Primary production is generally considered to be nitrogen limited when the Redfield ratio is below 16, but since ratios can vary between species and water bodies^26^ we have in this study not used 16 as a hard threshold between the limiting nutrients but rather as an indication, with ratios between 12 and 20 indicating a system in balance and ratios below or above this indicating nitrogen and phosphorus limitation respectively.

**Fig. 2:**
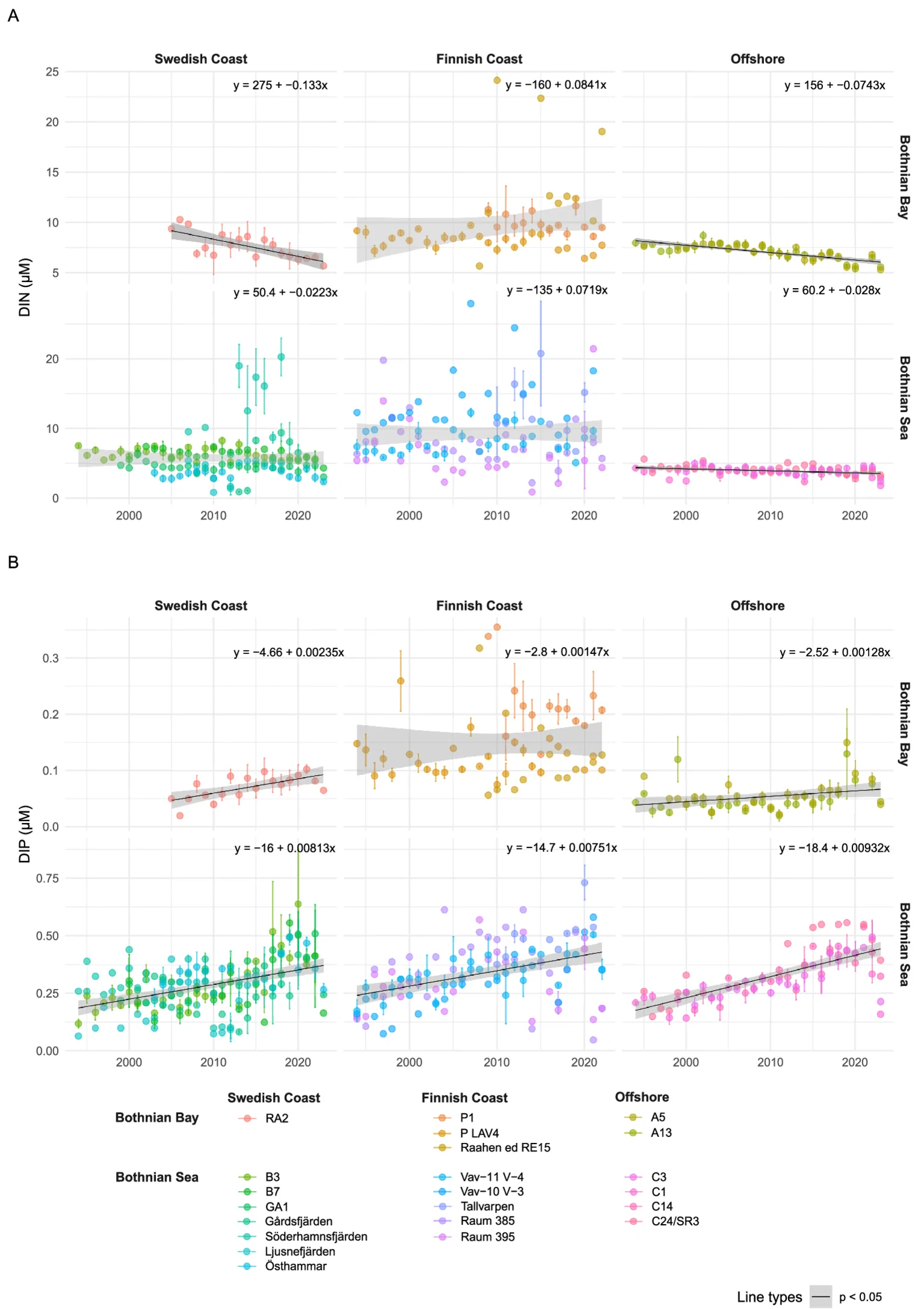
Winter surface concentrations of DIN (**a**) and DIP (**b**) (mean ± SE) in offshore and coastal areas in the Bothnian Bay and Bothnian Sea during the past 30 years. Changes over time with corresponding linear regression equation and confidence interval showing standard error. Significant trends according to Mann Kendall (*p* < 0.05) are indicated with solid lines.

Data were investigated for normality, and Mann-Kendall non-parametric tests were used for time series analysis, performed with MULTITEST, a VBA macro in Excel developed by Anders Grimvall, Claudia Libiseller, and Karl Wahlin (http://www.miljostatistik.se/mannkendall.html), Lindköping University. Correction for serial correlation was set to 1. Autocorrelation was checked in SPSS (Durbin-Watson test) and using Forcasting in SPSS showed ACP with lag 1 to be the most occurring. Programs used were primarily Excel (Microsoft), SPSS (IBM) and R for data handling and statistics. In addition, QGIS was used for plotting geographic information on DIN:DIP ratios along the Swedish and Finnish coasts. The map in Fig. 1 was made using R (https://cran.r-project.org/bin/windows/base/old/4.6.1/) and the following R packages: tidyverse version 2.0.0^27^, dplyr version 1.2.1^28^, ggplot2 version 4.0.3^29^, rnaturalearth version 1.2.0^30^, rnaturalearthdata version 1.0.0^31^, ggspatial version 1.1.10. ^32^. Inkscape version 1.4.4 (inkscape.org) was used to adjust legends within Fig. 2.

### Nutrient addition experiment

Since the Redfield ratio can vary depending on species and water bodies, a nutrient addition experiment was performed to evaluate its relevance in the investigated area. This was performed in a gradient from the central Bothnian Bay to the central Bothnian Sea, an area where the DIN:DIP ratios show a large spatial variation (dark blue dots in Fig. 1, stations A13, A19, A19-S, B1-N, B1, B1-S1, B1-S2, B5-N, B5 and C3, coordinates according to supplementary Table 1). Water was collected at 5 m for 10 stations using the Ferry-box system from the vessel KBV 181, 26–27 September 2023. The water was filtered through 200 μm to remove large zooplankton. The concentrations of nutrients (DIN, DIP and silicate) and chlorophyll *a* were measured at all stations. The samples were then incubated in a laboratory experiment to assess limiting nutrients, where chlorophyll concentration was used as a proxy for phytoplankton growth.

For each station, 300 ml of seawater were incubated in 5 different treatments, each having three replicates: (1) control (no addition of nutrients), (2) addition of nitrogen (addition of 11 µM DIN), (3) addition of phosphorus (addition of 0.6 µM DIP), (4) addition of silicon (addition of 22 µM silicate) and (5) addition of nitrogen, phosphorus and silicon (NPS, addition of 11 µM N, 0.6 µM P and 22 µM Si). The samples were incubated for 3 days at 15 °C with a light:darkness cycle of 12 h, and a light intensity of 70 µmol photon.m^− 2^.s^− 1^. The nutrients concentrations targeted by the additions resulted from a compromise in maximum DIN, DIP and silicate concentrations in the Bothnian Bay or Bothnian Sea, a DIN:DIP ratio close to Redfield and literature^11,15^. At the end of the experiment, the chlorophyll concentration was measured. The difference in chlorophyll concentration between the treatment and the control at the end of the experiment was used as response variable to evaluate the treatment response.

To measure the chlorophyll concentration, 100 ml of sample water were filtered through GF/F filters. The filters were extracted in 96% ethanol in the dark at 4°C overnight. The extracts were analyzed using a Perkin Elmer LS 30 fluorometer (433/673 nm excitation/emission wavelengths).

The single nutrient addition that gave the highest chlorophyll concentration was judged to be the most limiting substance. If the addition of another nutrient also had some positive effect on chlorophyll concentration, this was considered co-limitation^33^. The results were compared with the DIN:DIP ratio at the different sampling stations.

## Results

### Temporal trends in offshore and coastal areas

Dissolved inorganic nitrogen shows a decreasing trend in offshore areas of both the Bothnian Bay and the Bothnian Sea during the past 20 years (Fig. 2a). In the coastal areas, the conditions are more heterogenous, with a decreasing trend in the Swedish coastal waters of the Bothnian Bay, and no significant trend at the other coastal areas (Fig. 2a). The concentration of phosphate (DIP) shows an increasing trend in all the investigated areas except the Finnish Bothnian Bay coast. The increase being most pronounced in the offshore Bothnian Sea, where the concentration has approximately doubled from 1994 (0.21 ± 0.009) to 2022 (0.43 ± 0.07) (Fig. 2b). In the same area, DIN has decreased by about 10% (p-value 0.0041) from 1994 (4.32 ± 0.31) to 2022 (3.86 ± 0.65).

**Fig. 3:**
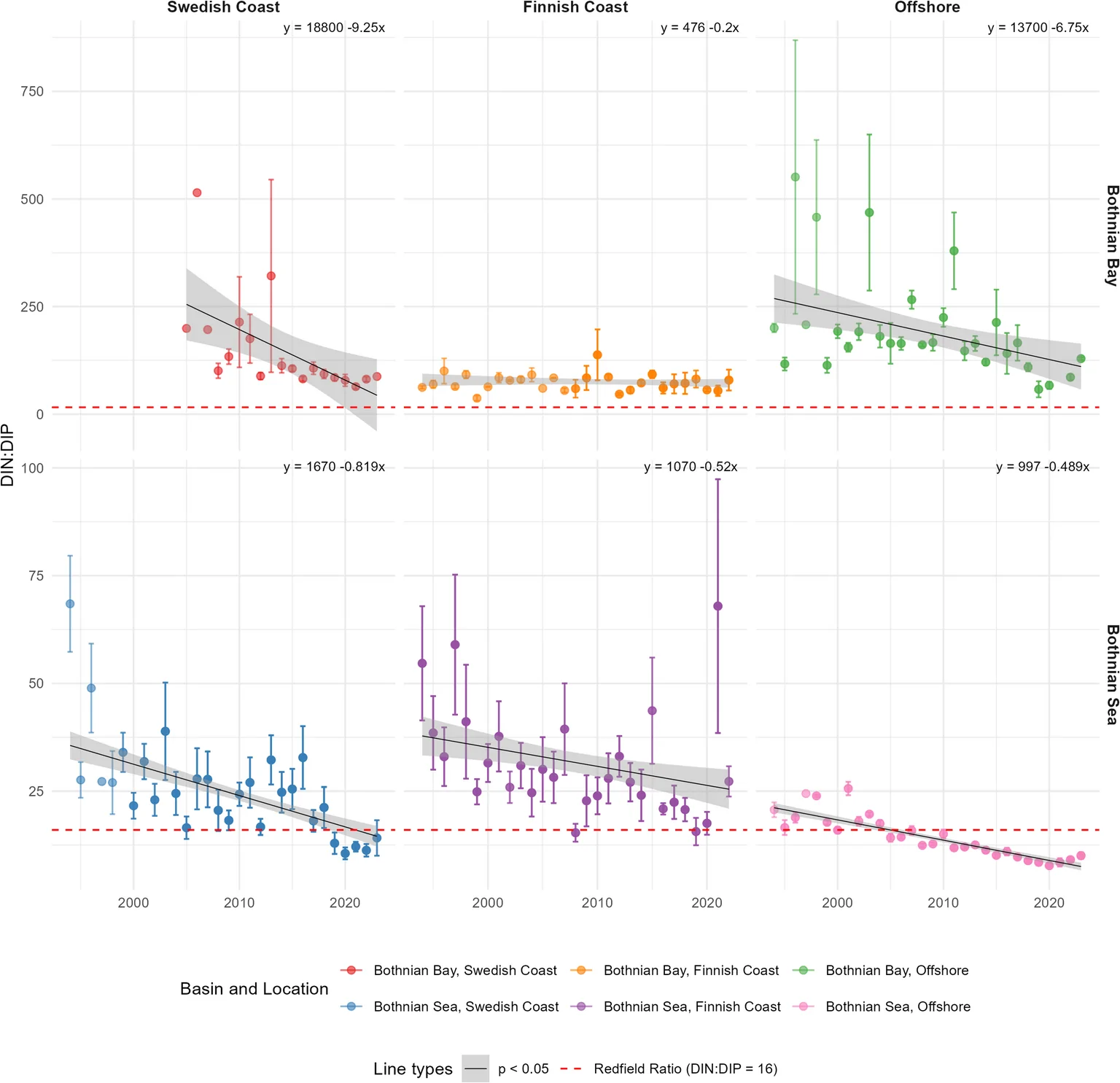
Winter surface waters DIN:DIP ratios (mean ± SE) in offshore and coastal areas in the Bothnian Bay and Bothnian Sea. Changes over time with corresponding linear regression equation and standard error as confidence interval. Significant trends according to Mann Kendall (*p* < 0.05) are indicated with solid lines. Redfield ratio = 16 indicated in red dotted line.

The DIN:DIP ratio calculated on winter values has decreased over time in all studied areas (Fig. 3). For the offshore areas, this means that the Bothnian Sea’s surface water has changed from being phosphorus-limited to being nitrogen-limited, a change that took place shortly after the turn of the Millennium. The Bothnian Bay, on the other hand, is still phosphorus-limited, despite a clearly declining DIN:DIP ratio. All trends in the open sea are significant. For the coast, the overall picture is like the open sea, with decreasing trends of the DIN:DIP ratio in all areas except the Finnish coast in the Bothnian Bay (Fig. 3).

### Relevance of Redfield ratio and limiting nutrient situation across the northern Quark

The nutrient addition experiment was performed on samples collected across a north-south gradient in the northern Quark. The chlorophyll *a* concentration of the samples varied from 2.4 to 4.4 µg l^− 1^, where no geographical upward or downward trend could be seen. In the subsequent laboratory experiment, the control treatment without any addition showed both decreasing and increasing chlorophyll levels in different samples (Supplementary Figure S.1), which can be explained by the interaction between nutrient restriction and the phytoplankton community’s initial biomass.

**Fig. 4:**
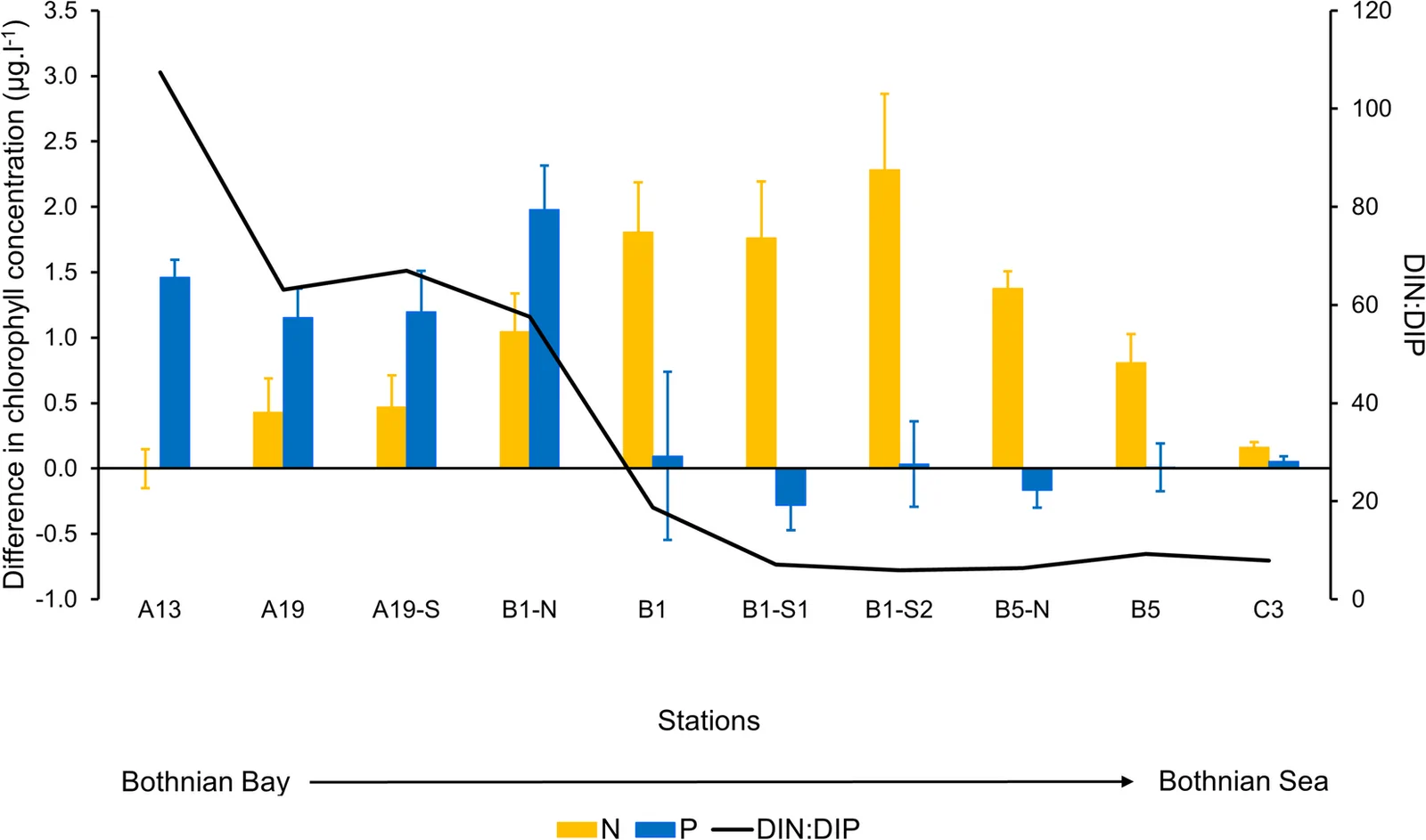
Response to nitrogen and phosphorus additions: difference in chlorophyll *a* concentration (addition - control) at the end of incubation (mean value ± SD) at the different stations. The SD of the difference in chlorophyll concentration was calculated using the square root of the sum of the squared standard deviations divided by the number of replicates of the control and the addition. The black line shows the DIN:DIP ratios at the sampling stations.

The experiment never showed that Si was a limiting nutrient (Supplementary Figure S.1), while N and P were limiting phytoplankton production at different stations (Fig. 4). At the northern stations (A13, A19, A19-S and B1-N), P-additions caused the largest chlorophyll increase, showing that phosphorus was the most limiting substance in the Bothnian Bay. However, nitrogen addition also gave a slight increase in chlorophyll concentration at DIN:DIP ratios up to 60, indicating co-limitation of both nitrogen and phosphorus in the area (stations A19, A19-S and B1-N). At the southern stations, nitrogen addition resulted in the highest chlorophyll increase, showing that this area was nitrogen limited. The observed cut-off point from phosphorus to nitrogen limitation was at the station with a DIN:DIP ratio of 18.7, i.e. slightly higher than the Redfield ratio (16). Phytoplankton composition data were only available for stations A13 and C3 (Supplementary Figure S.2). At A13, the northernmost station included in the experiment, the biomass of the mixotrophic ciliate *Mesodinium rubrum* dominated the phytoplankton community followed by flagellates from the groups Chrysophyceae, Cryptophyceae and Pyramimonadophyceae. At station C3 in the south, the nitrogen-fixing cyanobacterium *Aphanizomenon* sp. dominated with about 30% of the total biomass.

### Shifts in the distribution of different nutrient limitation groups

**Fig. 5:**
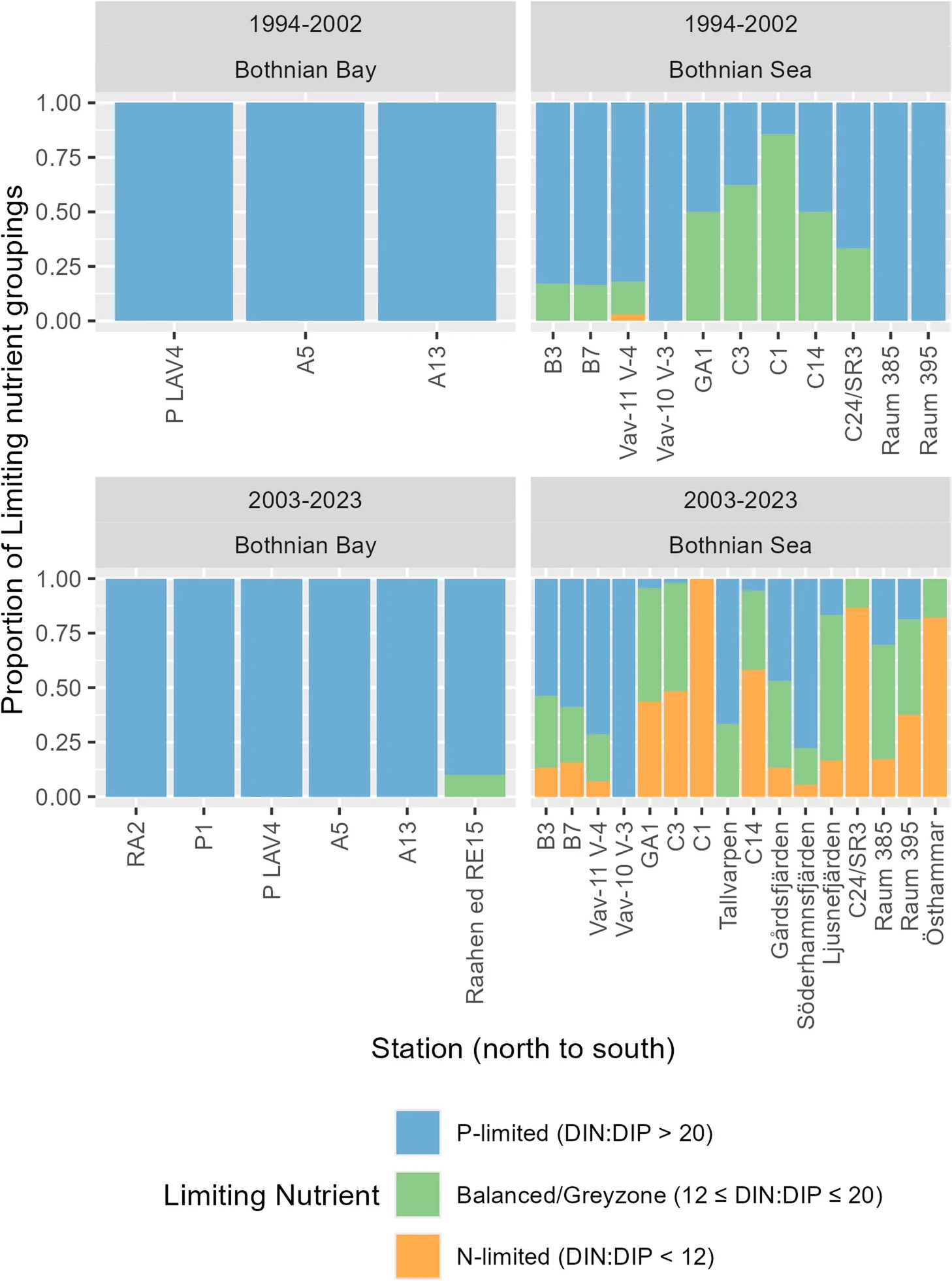
Proportion of nutrient limitation observations at stations in a north-south gradient (left-right) during 1994–2002 (upper panel) and 2003–2023 (lower panel). Limiting nutrients groupings according to: DIN:DIP > 20 phosphorus limitation (blue), DIN:DIP 12–20 balanced nutrient composition (green) and < 12 nitrogen limitation (orange). Please observe that there are more stations included after 2002.

Based on the results of the nutrient limitation experiment in combination with literature^15,25,26^, we classified the DIN:DIP ratios from winter (December-March) nutrient concentrations at all monitoring stations into different limitation groups: phosphorus limited (> 20), balanced (12–20) and nitrogen limited (< 12). Further, we aggregated the data into two time-periods, “past: before the Millennium shift” 1994–2002 and “present: after the Millenium shift” 2003–2023, to illustrate ecosystem changes over time (Fig. 5).

For the past period, the Bothnian Bay was 100% phosphorus-limited, while 10% of the observations in the northern Quark on the Swedish coast showed a balanced DIN:DIP ratio (Fig. 5). The offshore Bothnian Sea showed a more variable pattern, where ~ 50% of the samples were nutrient balanced and the rest phosphorus limited. This pattern had to a large extent changed until the present period, when only the Bothnian Bay remained phosphorus limited. The northern Quark was 50% phosphorus limited, 25% nutrient balanced, and 25% nitrogen limited. Further south in the Bothnian Sea coast, the nitrogen limitation was even more pronounced, and in the offshore Bothnian Sea the phosphorus limitation was not present any longer, and the sea area was mainly nitrogen limited, for approximately 75% of the observations.

### Spatial pattern of DIN:DIP ratio over time

**Fig. 6:**
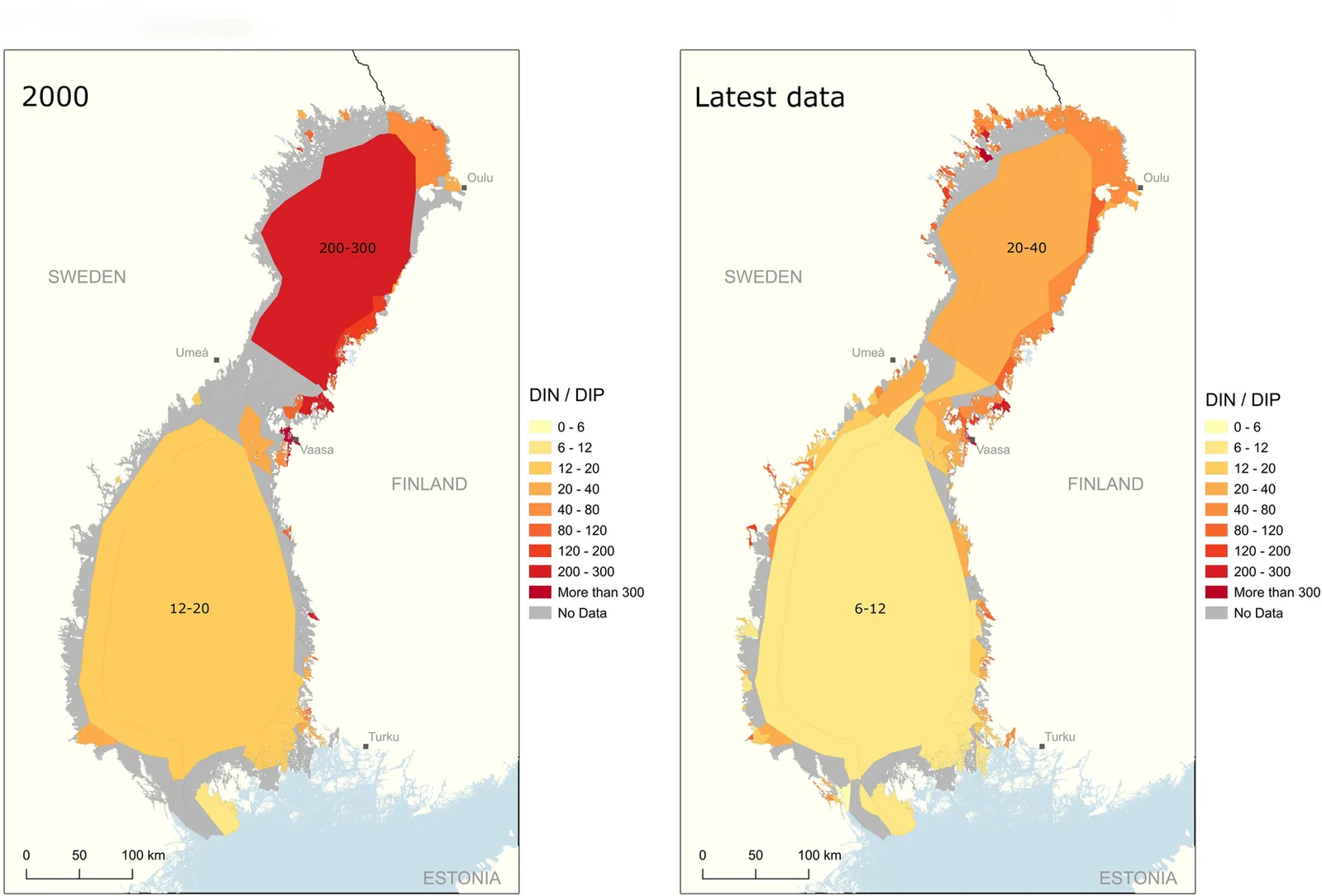
Classification of DIN:DIP ratios in different water bodies calculated from winter values (December-March). Compilation of data from year 2000 (left panel) and the most recent available year (right panel, from year 2005 and onwards, 70% are from 2018 or later). Generated in QGIS, version 3.30.2 https://qgis.org.

To further investigate the spatial distribution of the limiting nutrient, we categorized DIN:DIP ratios into 9 groups (DIN:DIP of 0–6, 6–12, 12–20, 20–40, 40–80, 80–120, 120–200, 200–300 and above 300) and made yearly calculations of DIN:DIP for all waterbodies in the Gulf of Bothnia with available data from year 2000 until 2022 (Fig. 6 and Supplementary Table S.2). Data from the beginning of this period (year 2000) show that the DIN:DIP ratios were high, 200–300, in the offshore Bothnian Bay and in some areas of the Finnish coast the ratio was a above 300, indicating strong phosphorus limitation (Fig. 6a). In the year 2000, the ratio varied between 20 and 40 in the offshore Bothnian Sea, indicating phosphorus limitation. The nutrient pattern changed consecutively through the investigated period (Supplementary Table S.2). Data for the most recent years (ca. 2020–2022) show that the predominant nutrient conditions in the Bothnian Sea year 2000 (DIN:DIP 20–40), now prevail in the Bothnian Bay (Fig. 6a, b). The offshore Bothnian Sea now shows nitrogen limitation, with a DIN:DIP ratio of 6–12. The coastal areas show a more heterogeneous distribution but also a general pattern of increase in nitrogen limitation with decreasing latitude both along the Swedish and Finnish coast in the end of the time series. (Fig. 6 and Supplementary Table S.2).

## Discussion

We show in this study that the nutrient levels have changed in both basins of the Gulf of Bothnia during the past 30 years. The phosphorus concentrations have generally increased in the whole area, with the highest increase observed in the Bothnian Sea. This increase cannot be explained by regional loadings to the area since the land-based loadings in the area have in fact decreased according to the latest HOLAS report^1^ or can be considered stable as shown by official Swedish statistics reports on nutrient loadings from the Swedish Agency for Marine and Water Management^34^. It is well-known that phosphorus is released from bottom sediments in anoxic marine environments and that this is also the case in the Baltic Sea^35,36^. The hypoxia situation of the Baltic Sea, especially in the Baltic Proper, is severe, and results from high nutrient loads from land in the Baltic region of the last century. The situation is worsened by the so-called “vicious circle”, where diazotrophic cyanobacteria promoted by the nitrogen limitation counteracts reductions of nitrogen loadings and maintains anoxic conditions in the deeper parts of the Baltic proper^37,38^,a phenomenon that is further worsened by climate change^39^.Oxygen concentrations are reported to decrease in the Bothnian Sea^20^, however neither the Bothnian Sea nor the Bothnian Bay currently have anoxic bottom waters in the offshore areas. In the Archipelago Sea, which we, in this study, have included in the Southern Quark area just south of the Bothnian Sea, the internal phosphorus loading, measured as increase of total phosphorus over the biological production season, accounts for as much as 50% of the phosphorus stock in the surface water layer^40^.

Previous investigations have shown, using primarily salinity to link the water masses, that the deep water of the Bothnian Sea is recruited from an intermediate layer at about 60 m depth in the Baltic Proper by northward transport over the sills of the Southern Quark^10,20^. Since the Baltic Proper contain higher nutrient levels than the Bothnian Sea it is thus likely that the increased phosphate concentrations in the Bothnian Sea is due to water inflow from the northern Baltic Proper.

At the beginning of the studied period, thirty years ago, the Bothnian Sea was phosphorus limited or nutrient balanced with DIN:DIP ratios ranging between 12 and 20. The recent data show that this basin is to a high degree nitrogen limited (DIN:DIP ratios 6–12) since the beginning of the 2020s, while the Bothnian Bay is still mostly phosphorus limited. The results of the nutrient addition experiment support these findings with phosphorus being the most limiting nutrient in the offshore Bothnian Bay and nitrogen the limiting nutrient in the offshore Bothnian Sea. The DIN:DIP ratio transition from phosphorus to nitrogen limitation at the time of the experiment occurred around 18–19, i.e. slightly higher than the classic Redfield ratio of 16. This falls within the expected range due to local variations addressed in the literature, where a shift between nitrogen and phosphorus limitation is suggested to occur for DIN:DIP ratios between 15 and 30^26^.

The present-day GIS map of DIN:DIP ratios shows that the entire offshore Bothnian Bay experienced a decrease in DIN:DIP ratios since 2000 and is now showing DIN:DIP ratios between 20 and 40 (Fig. 6b). This can still be considered phosphorus limitation but is closer to a balanced situation than with DIN:DIP ratios over 100 at the beginning of the time series. The imbalanced situation of pronounced phosphorus limitation, with DIN:DIP ratios > 100, resulted in low primary productivity in the Bothnian Bay^41^. With increased phosphorus concentrations and a more balanced nutrient situation one could expect an increase in primary productivity. However, inputs of riverine allochthonous organic matter, projected to increase in a warmer climate, have earlier been shown also to have a regulative impact on the ecosystem^42^ and need to be considered in combination with nutrients when discussing future implications of DIN:DIP changes in the area.

Generally, the results of this study are in line with earlier nutrient addition experiments in the area. Results from Andersson et al.^15^ show that at the beginning of 1990s two offshore stations located in the Bothnian Bay were phosphorus limited, while offshore stations in the Bothnian Sea were mainly nitrogen limited. However, Andersson et al.^15^ presented data indicating co-limitation between nitrogen, phosphorus and silica at all tested locations (offshore Bothnian Bay, Northern Quark and offshore Bothnian Sea) as additions of these nutrients induced increased phytoplankton growth. This differs from the present study where silica addition did not induce increased growth at any of the stations and phosphorus did not induce increased phytoplankton growth in the southern Northern Quark or in the Bothnian Sea. Silica has been increasing steadily in the area since the 1990s (shown by monitoring data), which explains why it is no longer a limitation for primary production. Our results are also in line with the results of similar nutrient addition experiments conducted on the Finnish side of the Gulf of Bothnia^11^, which also showed a clear phosphorus limitation in the Bothnian Bay in both winter and summer.

The use of bioassays to confirm general patterns of nutrient limitation indicated by DIN:DIP data analysis is a useful way to validate long-term monitoring data. The result of the bioassay in the present study clearly supports the use of DIN:DIP ratios to assess limiting nutrients in the Gulf of Bothnia. Using the DIN:DIP ratio of winter means is a common and accepted way to assess nutrient limitation and is for example the chosen method for the core indicators for inorganic nutrient and phosphorus used in the HELCOM assessment for eutrophication (^1^ and references to the core indicators therein). As a result of winter means being the most commonly use in monitoring, data on inorganic nutrients from the productive season are scarcer than for winter in the Gulf of Bothnia. In the latter years, available summer values show the overall pattern given by winter means with mainly nitrogen limitation in the Bothnian Sea and phosphorus limitation in the Bothnian Bay.

The DIN:DIP ratio both on the Swedish coast and in the offshore Bothnian Bay show a significant decrease over the past 25 years (Fig. 3). If the present trend continues, when prolonging the linear regression, a shift from a mainly phosphorus limited system to a nitrogen limited one may occur already in 10 to 40 years in the western coastal and offshore areas of the Bothnian Bay. On the Finnish coast of Bothnian Bay, the trend is different from the Swedish coast. Here, there is no significant upward trend for phosphorus (p-value 0.72), contrary to all other areas in this study, and also no decreasing trend of the DIN:DIP ratio. Why the coast of northern Finland differs so much from the other areas in the Gulf of Bothnia would be interesting to investigate further.

The consequences of a shift towards nitrogen limitation are primarily an increased risk of eutrophication, and its related symptoms, especially cyanobacterial blooms and growth of epiphytes. In a nitrogen limited environment, diazotrophic cyanobacteria will have an advantage over other phytoplankton species since they are able to assimilate atmospheric dissolved N_2_ in the water. Some nitrogen fixing cyanobacteria are also able to produce toxins, making the blooms a health issue in addition to environmental issues like excessive growth and oxygen depletion. Summer blooms of diazotrophic cyanobacteria have been common in the Baltic Proper since the 1960s^43^. These types of blooms are already seen in the Bothnian Sea, and they are reported to increase in duration and spatial coverage^18^,as well as increasing in terms of biomass^4,19^. Factors that will continue to favor diazotrophic cyanobacteria blooms in the Bothnian Sea going forwards are decreasing DIN:DIP ratios, as phosphorus increases, and increasing temperature^44–46^. At present, similar cyanobacteria blooms are less recurrent in the Bothnian Bay^4,47^. This difference in species composition of phytoplankton in phosphorus vs. nitrogen limited waters was also exemplified in the current nutrient addition experiment where the diazotrophic *Aphanizomenon* spp. dominated in the south, but not in the Bothnian Bay station at the same time.

The ability of the diazotrophic cyanobacteria to fix dissolved N_2_ in the water makes them contributors to nitrogen additions to the system. The cyanobacteria contribution to the nitrogen addition to the Bothnian Sea is still unknown but is estimated to exceed the external nitrogen loadings from rivers and atmosphere^48^. Increased nutrient levels may lead to worsening the eutrophication status. For the Gulf of Bothnia, recent assessments give warning that this is also topical here^1,17^. Decreasing oxygen levels in the Bothnian Sea are reported since 1994^20^ and increasing algal blooms may accelerate this due to the oxygen demand during the degradation phase. This may in turn lead to increased oxygen depletion in bottom waters and increased internal loading of phosphate, thus generating large areas of uninhabitable sea bottom as in the Baltic Proper.

In the Bothnian Bay the trends of decreasing DIN:DIP ratios, following the pattern already seen in the Bothnian Sea, should therefore work as an alarm clock. In fact, current models predict that despite the measures in place following the Baltic Sea Action Plan, the Bothnian Bay will not reach a good environmental status concerning eutrophication although most other basins in the Baltic Sea will^49^. We might expect blooms of diazotrophic filamentous cyanobacteria, decreasing oxygen levels and rewiring of the food web channeling also in the Bothnian Bay within a relatively short time. The forecasted increase of terrestrial organic matter loads^50^ and consequently decreased light in the Baltic Sea makes the situation difficult to predict and should be investigated further.

The results presented here emphasize that the perception of the Gulf of Bothnia as one coherent ecosystem not sensitive to nitrogen needs to be reconsidered. The two sea areas, the Bothnian Bay and the Bothnian Sea, have very different characteristics and should be discussed and dealt with separately in terms of management. The offshore Bothnian Sea has been nitrogen-limited for decades and, in addition, many coastal areas, at least in the southern parts, also show nitrogen limitation. This is due to both increasing phosphorus levels and decreasing nitrogen levels, which results in a greatly decreasing DIN:DIP ratio. The Bothnian Bay also shows a similar distinct declining trend of the DIN:DIP ratio in the offshore areas. The results of the addition experiment could implicate co-limitation between phosphorus and nitrogen takes place at DIN:DIP ratios up to 60, conditions that recent data show prevail in large parts of the Bothnian Bay. This would indicate that the Bothnian Bay is to some extent already nitrogen sensitive, and the addition of nitrogen to the area may thus have effects on the ecosystem. The decreasing DIN:DIP ratios indicate a possible upcoming shift to nitrogen limitation within a few decades; it is apparent that the area is sensitive to both phosphorus and nitrogen.

The increasing phosphorus levels in the Bothnian Sea are most likely due to inflow of phosphorus-containing water from the Baltic Proper^10,20^ causing the shift from phosphorus limitation to nitrogen limitation. This process is likely to continue if the problems with eutrophication and oxygen-depleted bottoms remain in the Baltic Proper, releasing phosphate that can be transported north to the Bothnian Sea. The nutrient situation in the Gulf of Bothnia is therefore influenced by the management of the Baltic Proper, decisions and remedies for the eutrophication situation in that basin will have a decisive effect on the Bothnian Sea and the Bothnian Bay. The influence of the Baltic Proper on the water quality in the Bothnian Sea also means that monitoring of the flow between the basins should be prioritized for management purposes. Increased knowledge of the magnitude of nutrient transport over the thresholds between the basins would be valuable in predicting future development of eutrophication issues. Similarly, the phosphorus levels are increasing in the Bothnian Bay without evidence of an increase in phosphate loadings from land^34^. This indicates that this basin is also influenced by phosphate-enriched water from more southerly basins, i.e., by inflow from the Bothnian Sea through the Northern Quark area. There is thus evidence to assume that the eutrophication problem in the Baltic Proper indirectly affects the Baltic’s northernmost sea basins. Both the Bothnian Sea and, by extension, the Bothnian Bay would benefit from an increased reduction in phosphorus discharges to the Baltic Proper. Additionally, recent studies have indicated that the oxycline in some of the basins of the Baltic Proper may be rising, which would have implications for the transport over the threshold into the Bothnian Sea^51^. If the oxycline reaches the halocline there is a risk of increased phosphorus transport over the threshold into the Bothnian Sea. These further stresses the influence of the situation in the Baltic Proper on the Gulf of Bothnia and its future development regarding the nutrient situation.

The findings presented here are relevant from a management perspective. The revision process of the European Wastewater Directive 2024/3019^21^ has sparked some debate, mainly due to a lack of sufficient data on nutrient dynamics and its consequences in the area. No general requirements were originally set, when the directive was first implemented, for nitrogen removal in northern Sweden and Finland. The expert panel working on the matter considered the Gulf of Bothnia to be unaffected by eutrophication and therefore the question of wastewater treatment was considered not to be relevant^52^. This should be reconsidered now that the conditions have changed. Governance authorities also need to consider the possible effect of nitrogen additions from diazotrophic cyanobacteria and whether reducing nitrogen in nitrogen limited areas can favor cyanobacteria and thus have a negative effect on the ecosystem. Although this has been raised as an issue research have clearly advised treating both for nitrogen and phosphorus^3,53^. Investigations performed in Swedish waters have shown positive effects of treating for both phosphorus and nitrogen^46^. However, wastewater is not the only source of nutrients, and most likely not the greatest, other sources of nutrients must also be considered and remedied if possible. From a governance perspective the view of the Gulf of Bothnia as a single entity with common, non-nitrogen and non-eutrophication sensitive characteristics, has despite previous studies^4,6,11^ proven difficult to change and is to some extent still prevalent in management considerations which should be changed.

In conclusion, it is evident that the eutrophication within the Baltic Sea area is still spreading, and it is also manifest that both the Bothnian Sea and the Bothnian Bay are either already nitrogen limited or heading towards that transition. This is primarily due to an increase in phosphate leading to decreasing DIN:DIP ratios. The consequences of increasing phosphate and a change in the nutrient ratio are increasing risk for future eutrophication in the Gulf of Bothnia. The water exchange between the Baltic basins is crucial for nutrient distribution, and its mechanisms require further investigation. While the two basins should be considered individually for management reasons, both the Bothnian Bay and the Bothnian Sea would benefit from decreasing input of both phosphorus and nitrogen.

## Supplementary Information

Below is the link to the electronic supplementary material.

Supplementary Material 1.

## Data Availability

The data used in this study are available from the Swedish national database SHARKweb (https://sharkweb.smhi.se/) provided by the Swedish Meteorological and Hydrological Institute (SMHI) and the Finnish database Merihavainnot.fi provided by the Finnish Environment Institute (SYKE). The datasets gathered and generated during the current study are available from the corresponding author on reasonable request. R-scripts and code can be found on Figshare (https://doi.org/10.6084/m9.figshare.33406147).
