# Supplementary material for: Rapid change of limiting nutrient in the Gulf of Bothnia, northern Baltic Sea
Source: Sci Rep. 2026 Sep 4;16:27798. doi: 10.1038/s41598-026-69219-6 (PMC13545236; doi:10.1038/s41598-026-69219-6)
Supplement: Supplementary file 1 [file 41598_2026_69219_MOESM1_ESM.pdf]

Supplementary material

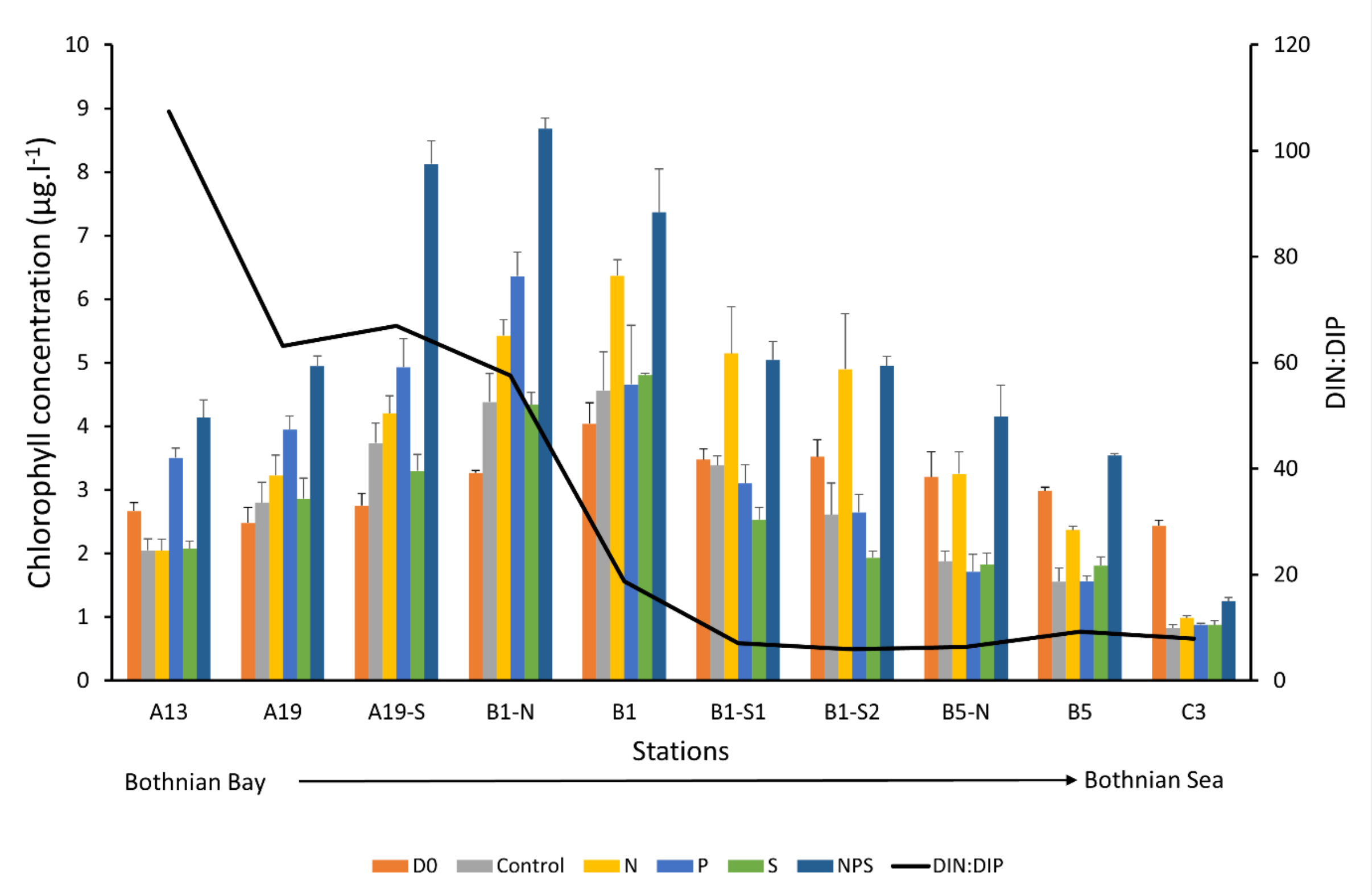

Supplementary Figure S.1. Chlorophyll concentration (mean ±SD) on day 0 (D0) at the sampling stations and day 3 at the laboratory incubations. Control= no addition, N = nitrogen addition, P = phosphorus addition, S = silicon addition. A13, A19, A19-S, B1-N, B1,B1-S1, B1-S2, B5 and C3 are different sampling stations, ordered from north to south. The black line shows the DIN:DIP ratio at the sampling stations.

Figure made in excel

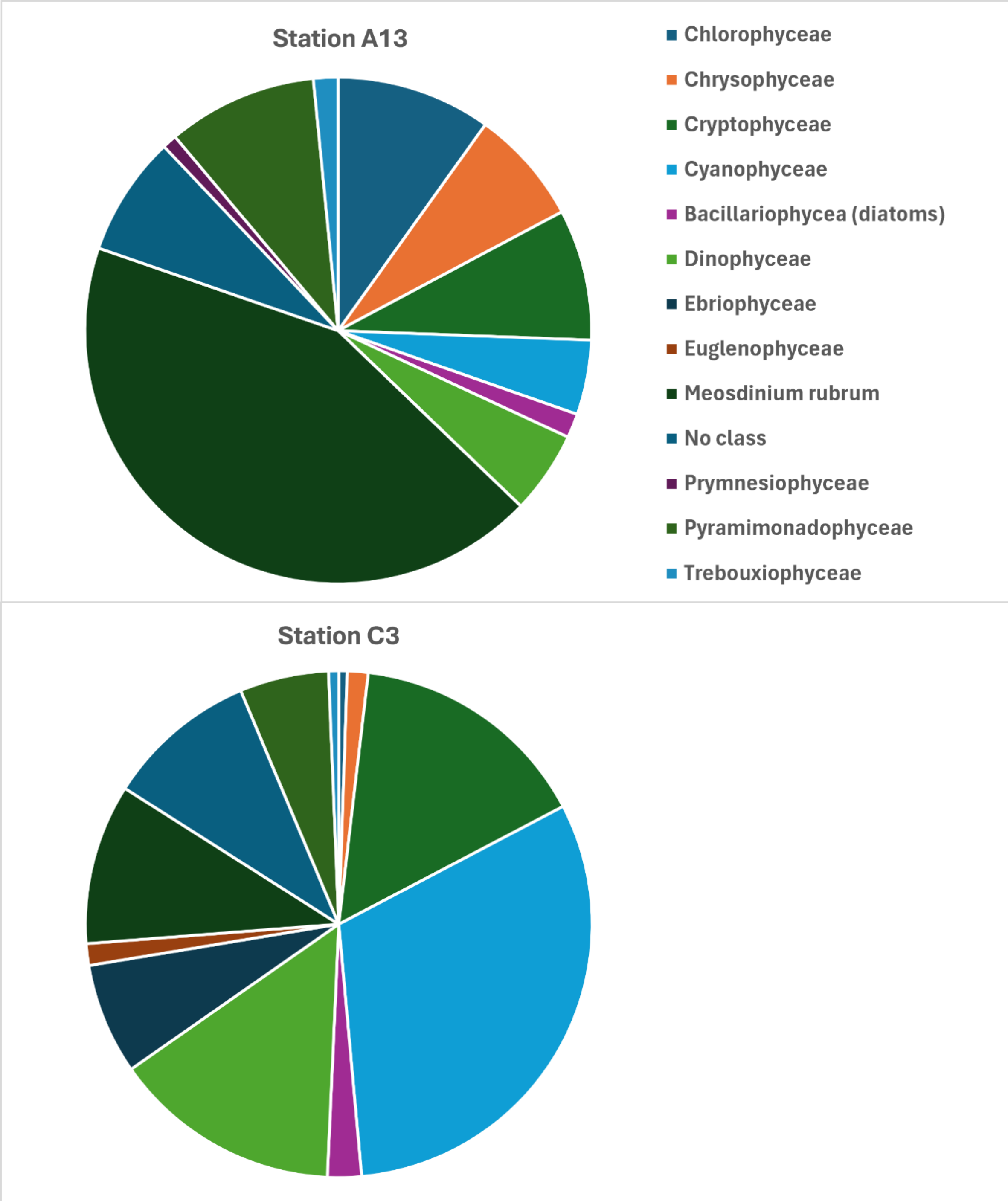

Supplementary Figure S.2. Phytoplankton composition analyzed from samples taken at the time of the sampling for the addition experiment, based on µg C/m3 for each Class of phytoplankton at stations A13 (upper) and C3 (lowest).

Figure made in excel

Supplementary Table S.1

Supplementary Table S.1. Station names and coordinates of the addition experiment.

Station	Degrees (°N)	Minutes (')	Degrees (°E)	Minutes (')
A13	64	42,50	22	04,00
A19	63	47,47	21	29,01
A19-S	63	42,47	21	17,82
B1-N	63	37,09	21	05,89
B1	63	31,54	20	53,30
B1-S1	63	30,12	20	44,09
B1-S2	63	28,11	20	35,00
B5-N	63	23,30	20	26,20
B5	63	18,55	20	16,82
C3	62	39,17	18	57,14

Supplementary Table S.2 DIN:DIP values from 2000 and most recent available DIN:DIP values in Swedish and Finnish waterbodies in the Gulf of Bothnia.

Code	DIN:DIP
	0-6
	6-12
	12-20
	20-40
	40-80
	80-120
	120-200
	200->

Waterbody name	DIN:DIP year 2000	Most recent data	Most recent DIN:DIP	Code
Torniofjärden sek namn				
Haparandafjärden sek namn		2022	67	
Katajafjärden		2022	67	
Knivskärsfjärden		2022	65	
Skomakarfjärden		2022	72	
Hamnskärsfjärden		2022	60	
Enskärsfjärden		2022	63	
Seskaröfjärden		2022	69	
S. Seskaröfjärden sek namn				

**Waterbody name**	**DIN:DIP year 2000**	**Most recent data**	**Most recent DIN:DIP**	**Code**
Kroksfjärden		2022	51	
Bodöfjärden		2022	75	
Repskärsfjärden	54	2005	54	
Norrbottens skärgårds kustvatten				
Törefjärden		2022	72	
Siknäsfjärden		2022	84	
Rånöfjärden		2022	35	
Slumpfjärden		2022	126	
Storöfjärden		2022	82	
Båtöfjärden				
Bergöfjärden		2022	74	
Rånefjärden	36	2022	59	
Gussöfjärden		2022	80	
N. Sigfridsöfjärden		2022	80	
Tistersöfjärden		2022	86	
S. Sigfridsöfjärden		2022	79	
Fjuksöfjärden	116	2008	300	
Bergnäsfjärden		2022	138	
Björköfjärden		2022	85	
Hamnöfjärden				
Granöfjärden		2022	181	
Mulöviken		2022	237	
Hindersöfjärden				
Saxskärsfjärden		2022	85	
Degeröfjärden		2022	79	
Bastaskärsfjärden				
Sörbrändöfjärden		2009	415	
Inre Lulefjärden		2009	58	
Yttre Lulefjärden		2009	57	
Sandöfjärden	148	2011	94	
Västantillfjärden		2022	78	
Germandöfjärden				
Börstskärsfjärden				
Måttsundsfjärden		2022	78	
Möröfjärden		2022	222	
Ersnäsfjärden		2022	86	
Sandgrönnfjärden				
Mannöfjärden		2022	72	
Holfjärden		2022	69	
Bastafjärden		2022	160	
Brändöfjärden		2022	157	
Harrbäcksfjärden		2022	82	
Storfjärden		2022	88	

Waterbody name	DIN:DIP year 2000	Most recent data	Most recent DIN:DIP	Code
Håkansöfjärden		2022	124	
Bärtnäsfjärden				
Rävahavet		2011	68	
Davids stenar				
Nördfjärden		2011	57	
Mjoöfjärden sek namn		2022	75	
Baggholmsdraget				
Haraholmsfjärden		2011	51	
Vargödraget		2021	59	
Inrefjärden		2021	86	
Yttrefjärden		2022	100	
Bondöfjärden		2013	173	
Part of Bothnian Bay open sea	282,5	2021	111	
N m Bothnian Bay coastal water				
Jävrefjärden		2022	93	
Bursfjärden		2022	92	
Kinnbäcksfjärden				
Åbyfjärden				
Tåmfjärden				
Byskefjärden		2015	81	
Rösnäsfjärden				
Bredviksfjärden				
Kågefjärden		2013	115	
Inre Kågefjärden		2011	111	
S m Bothnian Bay coast water				
Boviksfjärden		2013	173	
Hålfjärden				
Ursviksfjärden		2011	101	
Sörfjärden		2009	91	
Kurjoviken				
Skelleftehamnsfjärden				
Ytterviksfjärden		2022	123	
Simpan				
Skelleftebukten				
Burefjärden		2013	81	
Storsladan				
Sandvikssundet				
Bäckfjärden		2022	89	
Bjuröfjärden				
Southern Bothnian Bay coastal water				
Blackefjärden				

Waterbody name	DIN:DIP year 2000	Most recent data	Most recent DIN:DIP	Code
Kallviken				
Inre Lövselefjärden				
Yttre Lövselefjärden		2013	84	
Gumbodafjärden		2022	91	
Vändskärsfjärden				
Sikeåfjärden				
Ricklefjärden		2022	78	
Bygdefjärden		2022	58	
Part of Northern Quark open sea		2022	113	
Part of N n Northern Quark open sea				
Part of S n Northern Quark open sea				
N n Northern Quark coastal water				
Ostnäsfjärden		2013	95	
Ytterbodafjärden				
Lillfjärden		2011	113	
Täftefjärden		2009	54	
Yttre Täftefjärden				
Tavlefjärden		2013	56	
Raggavaviken		2013	155	
Österlångslädan		2013	75	
Inre Österfjärden		2010	90	
Holmsund		2021	109	
Yttre Österfjärden		2021	108	
Västerfjärden		2013	55	
Fjärdgrundsområdet sek namn		2021	27	
Mjölefjärden				
Megrundsområdet		2013	47	
Hörnefors området sek namn		2011	52	
Örefjärden	18	2022	13	
S n Northern Quark coastal water		2021	24	
Part of S n Northern Quark open sea				
Nordmalingsfjärden		2021	15	
Yttre Nordmalingfjärden				
Avafjärden		2021	34	
Degerfjärden				
N Bothnian Sea coastal water		2011	20	
Husumbukten				

**Waterbody name**	**DIN:DIP year 2000**	**Most recent data**	**Most recent DIN:DIP**	**Code**
Sannafjärden				
Ulträfjärden				
Risöfjärden				
Ällöviken				
Idbyfjärden		2016	26	
Örnsköldsviksfjärden		2018	19	
Dekarsöfjärden		2020	3	
Nötbolandsfjärden		2021	80	
Havsfjärden sek namn		2019	13	
Tennviken				
N High Coast coastal water		2011	16	
Bäckfjärden		2016	21	
Åvikfjärden				
Gullviksfjärden sek namn		2019	11	
Nätrafjärden		2021	13	
Näskefjärden		2011	21	
Mjältöfjärden sek namn		2021	13	
Norrfjärden		2019	10	
Dockstafjärden		2019	9	
Ullångersfjärden		2021	58	
Omnefjärden		2019	10	
Edsätterfjärden				
Gaviksfjärden	17	2021	11	
Sörleviken		2016	27	
Yttre Gaviksfjärden				
Kramforsfjärden sek namn		2009	99	
Bollstafjärden		2015	90	
Ramöfjärden sek namn				
Norafjärden		2016	30	
Grönsviksfjärden				
Storfjärden		2009	48	
Hemsösundet sek namn		2009	68	
Älandsfjärden		2019	10	
Norra sundet		2009	18	
S High Coast coastal water		2013	52	
Södra Sundet		2009	32	
Åvikebukten				
Klingerfjärden		2013	126	
Inre Tynderösundet		2021	15	
Alnösundet		2013	117	
Sundsvallsbukten				

Waterbody name	DIN:DIP year 2000	Most recent data	Most recent DIN:DIP	Code
Sundsvallsfjärden				
Draget		2014	121	
Svartviksfjärden		2013	76	
Juniskär-Bergsfjärden				
Björköfjärden				
Salen				
Yttre Björköfjärden				
Lubban				
Part of Bothnian Sea open sea	14,5	2021	10	
N M Bothnian Sea coastal water				
Part of Bothnian Sea open sea	15,9	2021	9	
Hudiksvallsfjärden		2020	19	
Agöfjärden sek namn		2020	10	
Gårdsfjärden		2020	14	
Njutångersfjärden		2020	12	
Siviksfjärden		2020	8	
Hålsängesfjärden				
Enångersfjärden		2020	7	
N S M Bothnian Sea coastal water				
Långvindsfjärden				
Skärsåfjärden sek namn				
Midsommarfjärden				
Söderhamnsfjärden		2018	87	
Sandarnesfjärden sek namn		2018	28	
Vallviksfjärden sek namn		2018	10	
Ljusnefjärden		2018	13	
Kusöfjärden sek namn				
Axmarfjärden				
Norrsundet		2017	17	
S S M Bottenhavets kustvatten				
Fårholmen				
Hamnskär				
Iggösundet				
Hilleviksfjärden		2011	21	
Harkskärsfjärden		2011	11	
Skutskärsfjärden sek namn	24	2012	18	
Yttre Fjärden		2017	109	
Avan				

Waterbody name	DIN:DIP year 2000	Most recent data	Most recent DIN:DIP	Code
Inre Fjärden				
Gävlebuktens utsjövatten	23	2012	21	
Lövstabukten				
Karlholmsfjärden				
Part of Bothnian Sea open sea	15,1	2022	10	
Öregrunds kustvatten				
Öregrundsgrepen				
Gällfjärden				
Kallriga Fjärden				
Ängsfjärden sek namn		2021	31	
Östhammarfjärden sek namn		2021	63	
Kasfjärden sek namn				
Östhammars kustvatten		2022	3	
Sandikafjärden				
Raggaröfjärden				
Norrfjärden				
Dragsfjärden				
Mjölkfjärden				
Norra Hargsviken				
Galtfjärden		2021	71	
Järsjöviken				
Edeboviken		2017	14	
Ortalaviken		2021	43	
Part of N Åland Sea open sea		2021	7	
Part of N Åland Sea open sea		2022	9	
Singöfjärden		2013	17	
Hargsviken				
Grisslehamn				
Kalajoki - Pyhäjoki	223,3	2017	60	
Luodonselkä		2018	59	
Kempeleenlahti		2018	34	
Liminganlahti		2018	28	
Hailuoto - Kuivaniemi	63,5	2022	57	
Rahjan saaristo pohjoinen		2022	79	
Raahe - Hailuoto		2022	85	
Santosenkari - Kattilankalla		2018	66	
Oulun edusta	24,7	2019	45	
Raahen edusta		2022	107	

Waterbody name	DIN:DIP year 2000	Most recent data	Most recent DIN:DIP	Code
Halttula - Kuivaniemi - Onkalo		2022	29	
Kuivaniemen ulompi edusta	165,9	2019	37	
Kyrönjoen edusta		2022	57	
Hankmo-Värlax				
Skinnarfjärden-Köklotfjärden		2021	134	
Revöfjärden		2021	88	
Bastufjärden (Söderudden)		2021	40	
Högskärsviken				
Gerby-Västervik-Iskmo	2238,1	2019	264	
Eteläinen kaupunginlahti-Varisselkä	2360,6	2019	579	
Sundomin sisäsaaristo	884,5	2019	120	
Stenskärsfjärden	298,8	2018	40	
Bergöfjärden	74,3	2021	32	
Halsön sisäsaaristo				
Korshamnsfjärden-Storfjärden	1200,4	2019	55	
Sommarösund				
Östra gloppet	259,4	2022	49	
Mickelsörarna-Rödgrynnorna	87,7	2021	67	
Replotfjärden		2018	116	
Ritgrund-Norra gloppet				
Valsörsgloppet		2008	28	
Utgrynnan-Molpehällorna	29,3	2021	19	
Gloppet		2019	31	
Bergö-Halsö		2021	23	
Rahjan saaristo etelä				
Lohtajanselkä				
Lohtaja-Kälviä		2021	143	
Kälviä-Kokkola	50,6	2007	32	
Kokkolan edusta	80	2019	46	
Luodon saaristo	137,8	2019	40	
Pietarsaaren edusta	174,2	2019	70	
Hästbådafjärden	123,8	2021	55	
Andra sjön	98,2	2015	77	
Monäsviken		2021	117	
Himanka-Kokkola	140,4	2011	74	
Tankar	141,6	2021	52	
Kallan	137	2019	56	
Uusikaarlepyy ulko	203,4	2017	117	
Harrströmin saaristo		2021	27	
Norrnäs				

Waterbody name	DIN:DIP year 2000	Most recent data	Most recent DIN:DIP	Code
Österfjärden (Nämpnäs)				
Järvöfjärden		2020	20	
Närpesfjärden		2020	31	
Pjelaxfjärden	94,1	2021	38	
Kristiinankaupunki länsi				
Kristiinankaupunki itä				
Kristiinankaupunki etelä		2021	28	
Skaftungin edusta		2015	31	
Kilviken				
Siipyynniemi		2021	40	
Korsnäs-Kaldonskär				
Kaskinen-Kristiinankaupunki	94,6	2021	19	
Kaskinen-Siipyy		2020	20	
Monåfjärden-Kalotfjärden	260,1	2015	272	
Tornio ulko	57,3	2022	62	
Tornio sisä		2022	44	
Röyttä sisä		2022	42	
Kemi-Simo ulko	72,7	2022	57	
Simo sisä	42,4	2022	45	
Maksniemi sisä		2022	79	
Ajos sisä		2022	61	
Kemi sisä		2022	65	
Hakkenpää - Tuulvesi	33,7	2021	15	
Vehmassalmi - Himoistenperä				
Mynälahden ulko-osa		2021	43	
Mynälahden sisäosa		2021	72	
Kustavin pohjoispuolinen ulkosaaristoalue	21,9	2021	16	
Iniön - Kustavin ulkosaaristoalue		2013	9	
Ströömi				
Kustavin lännenpuoli				
Merikarvian pohjoisosan sisäsaaristo				
Merikarvian edustan saaristo				
Peipunlahti				
Pokrunninlahti - Keikvesi				
Gummandooran saaristo		2014	35	
Baablinginlahti		2014	77	
Kuuskarinselkä				
Reposaaren - Outoorin alue		2014	28	
Eteläselkä				

Waterbody name	DIN:DIP year 2000	Most recent data	Most recent DIN:DIP	Code
Pihlavanlahti - Kolpanlahti	204,5	2014	106	
Preiviikinlahti - Viasvedenlahti				
Luvian ulkosaaristo				
Luvian sisäsaaristo				
Rauman ja Eurajoen saaristo	37,9	2022	22	
Eurajoensalmi	114,1	2022	94	
Olkiluodonvesi - Haapasaarenvesi		2022	113	
Merirauma - Nurmes				
Rauman edusta	31,8	2022	33	
Pyhämaan saaristo				
Mannervesi				
Ruotsinvesi - Velhovesi				
Liesluodon - Korsaaren edusta	30,4	2022	21	
Hylkimyksenaukko	71,2	2022	35	
Uudenkaupungin edusta	101,6	2022	69	
Lautvesi		2022	64	
Merikarvian avomeri				
Porin avomeri		2015	12	
Rahja - Kalajoki - Yppäri	31,9	2022	43	
Vaaranlahti - Pyhäjoki - Siniluoto				
Siniluodonlahti				
Kuljunlahti		2019	34	
Olkijoki - Siikajoki - Säärenperä		2005	21	
Haukipudas - Martinniemi - Räinänlahti	49,8	2018	73	
Ii - Olhava - Taipale				
Kihdin pohjoispuoli				
Muntinsalmi				
Luvian - Rauman avomeri		2022	12	
Uudenkaupungin avomeri	15,2	2022	7	
Northern Åland Sea				
Southern Åland Sea	9,7	2022	8	
Kyrksundet				
Finbofjärden				
Svartnöfjärden				
Marsund Norra				
Marsund Södra				
Bovik				
Ässkärsfjärden				

Waterbody name	DIN:DIP year 2000	Most recent data	Most recent DIN:DIP	Code
Sandviksfjärden				
Andersöfjärden				
Snäcköfjärden				
Västerfjärden Dånö				
Koxnan				
Bonäsfjärden				
Kalvfjärden				
Pantsarnäsfjärden				
Ivarskärsfjärden				
Bodafjärden				
Röjsbölefjärden				
Orrfjärden (m. Grundfjärden)				
Lillfjärden				
Vandöfjärden				
Ödkarbyviken				
Saltviksfjärden				
Färjsundet Norra				
Kornäsfjärden				
Simskälafjärden				
Engrundsfjärden				
Flatöfjärden				
Saggöfjärden				
Boxöfjärden				
Södra Delet				
Brändö innerskärgård				
Ängskärsfjärden				
Skiftet Norra				
Norra Delet	13,1	2021	10	
